# Hemodialysis (HD) dose and ultrafiltration rate are associated with survival in pediatric and adolescent patients on chronic HD—a large observational study with follow-up to young adult age

**DOI:** 10.1007/s00467-021-04972-6

**Published:** 2021-03-02

**Authors:** Verena Gotta, Olivera Marsenic, Andrew Atkinson, Marc Pfister

**Affiliations:** 1 Pediatric Pharmacology and Pharmacometrics, University of Basel Children’s Hospital, Spitalstrasse 33, 4031 Basel, Switzerland; 2 Pediatric Nephrology, Stanford University School of Medicine, Lucile Packard Children’s Hospital, Stanford, CA USA; 3 Certara, Princeton, NJ USA

**Keywords:** Hemodialysis, Survival, Adequacy, Kt/V, Body surface area, Ultrafiltration

## Abstract

**Background:**

Hemodialysis (HD) dose targets and ultrafiltration rate (UFR) limits for pediatric patients on chronic HD are not known and are derived from adults (spKt/V>1.4 and <13 ml/kg/h). We aimed to characterize how delivered HD dose and UFR are associated with survival in a large cohort of patients who started HD in childhood.

**Methods:**

Retrospective analysis on a cohort of patients <30 years, on chronic HD since childhood (<19 years), having received thrice-weekly HD 2004–2016 in outpatient DaVita centers. Outcome: Survival while remaining on HD. Predictors: (I) primary analysis: mean delivered dialysis dose stratified as spKt/V ≤1.4/1.4–1.6/>1.6 (Kaplan–Meier analysis), (II) secondary analyses: UFR and alternative dialysis adequacy measures [eKt/V, body-surface normalized Kt/BSA] on continuous scale (Weibull regression model).

**Results:**

A total of 1780 patients were included (age at the start of HD: 0–12y: *n*=321, >12–18y: *n*=1459; median spKt/V=1.55, eKt/V=1.31, Kt/BSA=31.2 L/m^2^, UFR=10.6 mL/kg/h). (I) spKt/V<1.4 was associated with lower survival compared to spKt/V>1.4–1.6 (*P*<0.001, log-rank test), and spKt/V>1.6 (*P*<0.001), with 10-year survival of 69.3% (59.4–80.9%) versus 83.0% (76.8–89.8%) and 84.0% (79.6–88.5%), respectively. (II) Kt/BSA was a better predictor of survival than spKt/V or eKt/V. UFR was additionally associated with survival (*P*<0.001), with increased mortality <10/>18 mL/kg/h. Associations did not alter significantly following adjustment for demographic characteristics (age, etiology of kidney disease, and ethnicity).

**Conclusions:**

Our results suggest usefulness of targeting Kt/BSA>30 L/m^2^ for best long-term outcomes, corresponding to spKt/V>1.4 (>12 years) and >1.6 (<12 years). In contrast to adults, higher UFR of 10–18 ml/kg/h was not associated with greater mortality in this population.

**Supplementary Information:**

The online version contains supplementary material available at 10.1007/s00467-021-04972-6.

## Introduction

Hemodialysis (HD) dose targets for adults have been defined in terms of small solute clearance, currently considered the best measure of HD adequacy [1]. It is measured using urea kinetics during HD as reference solute, with a target of spKt/V > 1.4 and minimum of 1.2 defined (with sp = single pool urea distribution model, K = urea clearance, t = treatment time, V = urea distribution volume [2]). Studies in adults have shown a correlation between low spKt/V and increased mortality [3, 4], while higher than target spKt/V has not been associated with improved survival in adults [5]. Concerning fluid removal during HD, ultrafiltration rates (UFR) > 10–13 mL/kg/h (> 1.0–1.3% per kg per hour) have been associated with mortality in adults [6].

The significance of these findings remains unclear for children on chronic HD, as small patient numbers limit systematic clinical investigations in this population. Small solute clearance targets are generally adopted from adults in the absence of pediatric studies [7]. However, children may require higher spKt/V targets, due to higher urea rebound [8, 9] and a higher ratio of body surface area (BSA) to body weight compared to adults, linked to higher metabolic rate. Hence, alternative metrics of urea clearance may be favored, such as equilibrated eKt/V (eKt/V) or BSA-normalized Kt/BSA [10–13]. While well investigated in adults, the UFR effect on pediatric outcomes has not yet been studied despite concerns that high UFR increases the risk of cardiovascular mortality [6]. High UFR is not uncommon in children [14] and is driven by nutritional needs of growing pediatric HD patients. This may be worrisome [15] given that prescribed UFR is closely related to interdialytic weight gain (IDWG), which has been associated with left ventricular hypertrophy at IDWG > 4% [16] and pre-HD hypertension [17] in children. Yet, there are no studies of the UFR effect on outcomes in this population. In the absence of outcome data, fluid removal in children has been proposed to be limited to 1.5±0.5% of body weight per hour (max. 5% during one session) [18].

Here we report the first study investigating the relationship between HD dose, UFR, and survival in a large pediatric cohort on thrice-weekly HD [19]. Our objectives were to evaluate (I) the association between spKt/V (stratified as low: ≤1.4, target: 1.4–1.6, high: >1.6) and survival while remaining on HD, (II) to compare the association of different small solute clearance metrics (spKt/V, eKt/V, Kt/BSA) with survival on a continuous scale, and (III) to evaluate the effect of fluid balance indices (UFR and associated IDWG) on survival.

## Methods

### Study design and participants

Data used for this retrospective analysis originate from an observational cohort of patients who started chronic HD in childhood (≤ 19 years), with a maximum follow-up until < 30 years of age, having received standardized thrice-weekly HD between 05/2004 and 03/2016 in outpatient DaVita Kidney Care (DaVita Inc., Denver, CO, USA) dialysis centers [9, 14, 19]. The scientific use of the deidentified standardized electronic medical records was approved by DaVita (DaVita Clinical Research®, Minneapolis, MN); IRB approval was not required since retrospective analysis was performed on the deidentified dataset.

### Variables and measurement

Age at the start of dialysis was calculated from treatment and first dialysis date (age in years recorded as integers). Further recorded baseline demographic factors potentially associated with mortality in stage 5 chronic kidney disease (CKD 5) patients [20, 21] included gender, etiology of kidney disease (e.g., secondary glomerulonephritis), and comorbidities (e.g., diabetes and connective tissue disease).

Dialysis dose in terms of spKt/V and eKt/V was calculated for each recorded treatment [2, 22]. Three alternative dialysis adequacy markers normalized by weight^0.67^, weight^0.75^, and BSA, respectively (Kt/W^0.67^, Kt/W^0.75^, Kt/BSA), were calculated [13]:

$$ \mathrm{Kt}/{\mathrm{W}}^{0.67}=\mathrm{spKt}/\mathrm{V}\cdot {\mathrm{V}}_{\mathrm{TBW}}/{\mathrm{W}}^{0.67} $$

$$ \mathrm{Kt}/\mathrm{BSA}=\mathrm{spKt}/\mathrm{V}\cdot {\mathrm{V}}_{\mathrm{TBW}}/\mathrm{BSA} $$

where V_TBW_ = total body water (L) according to Cheek [23, 24] or Watson [25], respectively (assumed to equal urea distribution volume V), BSA = BSA according to Mosteller [26], and W = target dry weight (kg).

Fluid removal in terms of UFR (mL/kg/h) and total ultrafiltration (UF, % per kg dry weight per session) was derived for each treatment from pre- and postdialytic weight and treatment duration. Interdialytic weight gain (IDWG) was derived from post- and predialytic weight difference of subsequent treatments and was expressed as %increase per kg dry weight over 2 days [17].

For each patient, mean individual values of spKt/V, eKt/V, Kt/W^0.67^, Kt/W^0.75^, Kt/BSA, UFR, UF, and IDWG during follow-up on HD were calculated for further analysis; no minimum repeated observation number was defined for this calculation.

### Primary outcome

The primary outcome of interest was overall survival while remaining on HD (death from any cause) stratified by dialysis dose (spKt/V ≤ 1.4 / 1.4–1.6 / >1.6). As supplementary analyses, the same outcome was investigated within two age-groups at the start of dialysis (0–12 / > 12–19 years), and relative survival (hazard ratio) was investigated adjusted for age and other potentially relevant demographic characteristics (gender, etiology of kidney disease, ethnicity, and comorbidities), considering potential interactions with age at the start of HD.

### Secondary outcome

The secondary outcome of interest was absolute survival while remaining on HD as a continuous function of dialysis dose (measured as spKt/V, eKt/V, Kt/W^0.67^, Kt/W^0.75^, or Kt/BSA), fluid balance (UFR, UF, or IDWG), and other potentially relevant demographic characteristics, considering potential interactions with age at the start of HD.

### Statistical methods

Given the low number of patients with follow-up > 15 years and the maximal age during follow-up of 29 years (resulting in a maximal follow-up period of 10 years in patients starting dialysis with 19 years), later observations were censored at 15 years. The primary outcome analysis included all patients for whom duration of follow-up on HD was recorded with information on spKt/V and age, whereas supplementary and secondary outcome analyses were performed on complete-case data with respect to further potentially relevant demographic characteristics. Patients lost to follow-up (e.g., due to transplantation, ongoing HD treatments > 03/2016 (date of data extraction), discharge to other facility (details Table 1)) were censored at their last observation.

**Table 1:** Patient characteristics

Variable [*n* (%) or median (IQR)]	spKtV ≤ 1.4 (low)	spKt/V 1.4–1.6 (target)	spKt/V > 1.6 (high)	All patients (primary analysis)	Complete-cases
Total number of individuals	489	531	759	1780	1493
Age at dialysis start (years)	16.7 [15.0, 18.0]	16.3 [13.6, 18.0]	16.0 [12.7, 18.0]	16.3 [13.6,18.0]	16.2 [13.8, 18.0]
Target dry weight (kg)	65.9 [53.3, 82.4]	58.8 [48.7, 70.0]	50.7 [41.7, 58.9]	56.1 [45.3,68.5]	56.4 [46.1, 78.7]
Body surface area (m^2^, Mosteller)	1.75 [1.54, 2.00]	1.64 [1.47, 1.82]	1.48 [1.31, 1.63]	1.59 [1.40,1.79]	1.60 [1.41, 1.80]
Gender (male)	333 (68.1%)	332 (62.5%)	323 (42.6%)	988 (55.5%)	814 (54.5)
Time of follow-up on hemodialysis (years)	1.5 [0.4–3.7]	3.6 [1.3–6.7]	3.9 [1.6–7.8]	3.0 [1.1,6.5]	3.3 [1.3,7.0]
Ethnicity					
African	185 (37.8%)	212 (39.9%)	186 (24.5%)	583 (32.8%)	501 (33.6%)
Caucasian	160 (32.7%)	136 (25.6%)	244 (32.1%)	541 (30.4%)	434 (29.1%)
Hispanic	98 (20.0%)	137 (25.8%)	238 (31.4%)	473 (26.6%)	414 (27.7%)
Other^1^	46 (9.5%)	46 (8.6%)	91 (12.0%)	181 (10.2%)	143 (9.6%)
Etiology according to USRDS (%)					
Glomerulonephritis	117 (23.9%)	137 (25.8%)	205 (27.0%)	459 (30.4%)	454 (30.4)
Cystic/hereditary/congenital diseases	77 (15.7%)	88 (16.6%)	117 (15.4%)	282 (18.7%)	278 (18.6)
Secondary glomerulonephritis/vasculitis	61 (12.5%)	55 (10.4%)	101 (13.3%)	218 (14.5%)	214 (14.3)
Etiology uncertain	55 (11.2%)	62 (11.7%)	73 (9.6%)	190 (12.6%)	189 (12.7)
Hypertensive/large vessel disease	52 (10.6%)	50 (9.4%)	67 (8.8%)	169 (11.2%)	168 (11.3)
Interstitial nephritis/pyelonephritis	27 (5.5%)	41 (7.7%)	65 (8.6%)	133 (11.2%)	133 (8.9)
Miscellaneous conditions	3 (0.6%)	7 (1.3%)	16 (2.1%)	26 (1.7%)	26 (1.7)
Diabetes	5 (1.0%)	4 (0.8%)	8 (1.1%)	17 (1.1%)	17 (1.1)
Neoplasms/tumors	3 (0.6%)	7 (1.3%)	5 (0.5%)	14 (0.9%)	14 (0.9)
Not documented	89 (18.2%)	80 (15.1%)	103 (13.6%)	272 (15.3%)	-
Reasons for lost to follow-up (%)					
Death	40 (8.2%)	38 (7.2%)	71 (9.4%)	149 (8.4%)	138 (9.2)
Number deceased < 19 years (%)	15 (37.5%)	11 (28.9%)	23 (32.9%)	49 (32.9%)	42 (30.4%)
Transplant	179 (36.6%)	209 (39.4%)	238 (31.4%)	626 (35.2%)	559 (37.4%)
Ongoing HD treatment beyond time of data extraction	118 (24.1%)	185 (34.8%)	227 (29.9%)	530 (29.8%)	414 (27.7%)
Discharge to other facility	80 (16.4%)	60 (11.3%)	152 (20.0%)	292 (16.4%)	244 (16.3%)
Other	64 (13.1%)	32 (6.0%)	56 (7.4%)	153 (8.6%)	113 (7.6%)
Not documented	8 (1.6%)	7 (1.3%)	15 (2.0%)	30 (1.7%)	25 (1.7%)
Cause of death					
Cardiovascular	15 (37.5%)	14 (36.8%)	22 (31.0%)	51 (34.2%)	49 (35.5%)
Infectious	0	3 (7.9%)	6 (8.5%)	9 (6.0%)	9 (6.5%)
Other	9 (22.5%)	10 (26.3%)	16 (22.5%)	35 (23.5%)	30 (21.7%)
Unknown	16 (40.0%)	11 (28.9%)	27 (38.0%)	54 (36.2%)	50 (36.2%)
Comorbidities					
Diabetes	63 (12.8%)	91 (17.1%)	151 (19.9%)	305 (17.1%)	269 (18.0%)
Connective tissue disease	13 (2.7%)	22 (4.1%)	40 (5.2%)	75 (4.2%)	72 (4.8%)
Other	9 (1.8%)	21 (4.0%)	34 (4.5%)	64 (3.6%)	56 (3.8%)
Treatment related variables^2^					
spKt/V (-)	1.28 [1.17, 1.34]	1.50 [1.46, 1.55]	1.76 [1.67, 1.91]	1.55 [1.38, 1.73]	1.56 [1.39, 1.73]
eKt/V (-)	1.07 [0.99, 1.13]	1.26 [1.22, 1.31]	1.49 [1.41, 1.62]	1.31 [1.16, 1.46]	1.31 [1.17, 1.46]
Kt/W^0.67^ (L/kg^0.67^)	2.75 [2.46, 3.03]	3.24 [2.97, 3.52]	3.68 [3.38, 4.01]	3.30 [2.90, 3.68]	3.33 [2.92, 3.70]
Kt/BSA (L/m^2^)	26.3 [23.5, 28.5]	30.6 [28.3, 33.0]	34.6 [31.7, 37.4]	31.2 [27.4, 34.4]	31.4 [27.9, 34.7]
Ultrafiltration rate (mL/kg/h)	8.1 [5.3, 11.3]	10.6 [7.50, 14.0]	12.3 [8.7, 15.9]	10.6 [7.2, 14.2]	10.8 [7.4, 14.5]
Total ultrafiltration (%)*	2.7 [1.8, 3.6]	3.6 [2.6, 4.5]	4.0 [2.9, 5.0]	3.5 [2.4, 4.6]	3.6 [2.5, 4.7]
Interdialytic weight gain (%/2 day)	2.4 [1.6, 3.2]	3.1 [2.2, 3.9]	3.6 [2.5,4.5]	3.0 [2.0, 4.0]	3.1 [2.1, 4.1]

^1^*n* = 2 missing values in the overall population

^2^*n* = 1 with missing spKt/V, *n* = 3 with missing eKt/V, and *n* = 17 with missing interdialytic weight gain in the overall population

*USRDS* United States renal data system. Total ultrafiltration (%) = V_UF_ (L)/target dry weight (kg), with V_UF_ = ultrafiltration volume (L) calculated as predialytic weight (kg) – postdialytic weight (kg). Interdialytic weight gain (IDWG, %) = IDWG (kg)/target weight (kg), with IDWG (kg) = predialytic weight – weight aſter preceding HD (Marsenic et al. [17])

The primary outcome was investigated using nonparametric methods (Kaplan–Meier). The log-rank test was used to compare survival on HD between subgroups where Kaplan–Meier curves suggested a difference between any two of the spKt/V groups. Survival probabilities were derived with 95% confidence intervals at 5, 10, and 15 years. In a supplementary analysis of the primary outcome, Kaplan–Meier curves were generated for age groups < / > 12 years at the start of HD, and relative survival for the spkt/V strata was evaluated by fitting both the univariable and multivariable parametric Weibull proportional hazards models. Multivariable models were adjusted for potential confounders (demographic characteristics). The appropriateness of the Weibull distribution for the baseline hazard function was evaluated by comparing the predicted survival curve of the null model (without covariates) with the corresponding nonparametric Kaplan–Meier survival curve. The proportional hazard assumption was verified visually, using the log(time) vs. log(-log(survival(time)) plot and the Schoenfeld residuals.

Absolute survival as a continuous function of dialysis adequacy measures and fluid balance indices (secondary outcomes) was investigated by fitting a Weibull accelerated failure time model. Covariates were included in the adjusted model by considering linear, log-linear, quadratic, and cubic relationships. The best covariate relationship was chosen based on visual agreement of the corresponding predicted log hazard function with the one predicted using a smoothing spline, the Akaike information criterion, and *P* value from the likelihood ratio test (LRT) compared to the null model. Variables with a *P* value < 0.05 (LRT) in univariable analyses were further considered for the multivariable adjusted analysis. Among the variables referring to a metric of dialysis adequacy (spKt/V, eKt/V, Kt/W^0.67^, Kt/W^0.75^, Kt/BSA) or fluid balance (UFR, UF, IDWG), only the variable with the lowest *P* value was used for forward selection and backward deletion considering further demographic baseline characteristics.

Based on initial findings, the following post hoc subgroup and sensitivity analyses were performed. For the primary outcome: (a) censoring all patients at 19 years of age, (b) stratification of age group ≤ 12 years into < 6 years and 6–12 years at the start of HD; and (c) piece-wise analysis the first 2 years versus ≥ 2 years to investigate the potential magnitude of time-varying hazard (visual investigation suggested potential nonproportionality within the first 2–3 years, although the test for proportional hazards indicated no violation of this assumption). With regard to the secondary outcome: (a) censoring all patients at 19 years of age, (b) for covariates retained in the multivariate model, Kaplan–Meier curves were used as a goodness-of-fit comparison, stratifying the variable of interest into quartiles. (c) The correlation between Kt/BSA and spKt/V was investigated stratified by gender and age, to determine Kt/BSA values that would correspond to target spKt/V of 1.4–1.6 associated with good survival in adolescent patients. (d) Correlations between disease-related mortality risk factors [27] with investigated dialysis adequacy measures and fluid balance indices were investigated by the Spearman rank correlation coefficient.

Throughout a *P* value of less than 0.05 was considered statistically significant. All analyses, figures, and statistics were generated in R version 3.6.2.

## Results

### Patients and available predictor variables

From the 1852 patients with in total 53,903 treatment records with spKt/V evaluation (having mainly been performed monthly, median: 16, IQR: 5–43 per patient) previously reported [19], a total of 1780 patients (52,083 HD treatment records with spKt/V evaluation) had their duration and follow-up on HD documented for the primary analysis (*n* = 321 having started HD at 0–12 years of age, *n* = 1459 at > 12–19 years). Among these, 1493 (83.9%) had complete baseline characteristic data and were included in adjusted analyses (Table 1).

### Primary outcome

**Fig. 1:**
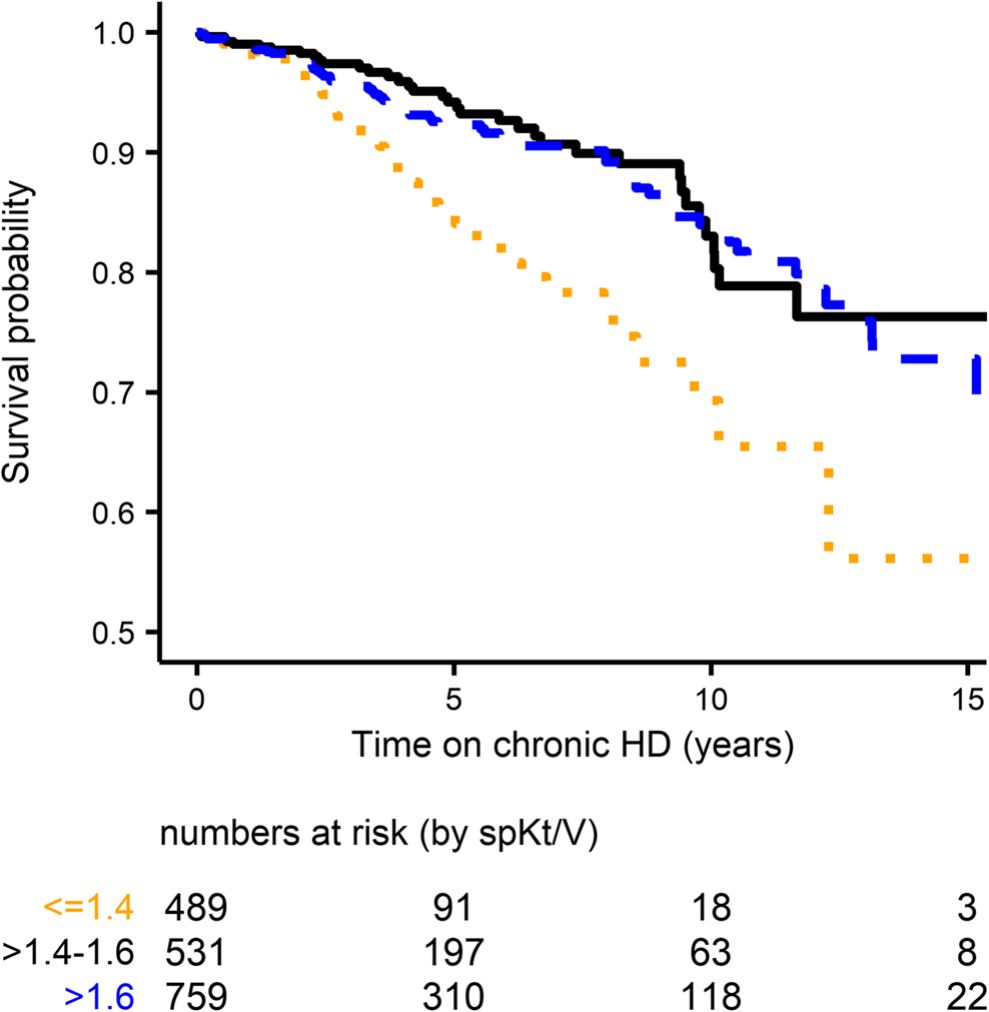
Survival probability while remaining on chronic hemodialysis (Kaplan–Meier curve) by mean spKt/V delivered (primary outcome); log-rank test: spKtV ≤ 1.4 (dotted) *versus* > 1.4–1.6 (solid): *P* = 0.0007. spKtV ≤ 1.4 versus > 1.6 (dahsed): *P* = 0.002

The Kaplan–Meier survival curve while remaining on HD stratified by mean delivered spKt/V is shown in Fig. 1 (primary endpoint). Survival was significantly lower in patients with low spKt/V ≤ 1.4 compared to patients with target spKt/V > 1.4–1.6 (log-rank test *P* < 0.001) and compared with high spKt/V > 1.6 (*P* = 0.002) but did not differ between patients treated with target versus high spKt/V (*P* = 0.5). Survival estimates at 5–15 years are provided in Table 2.

**Table 2:** Observed overall survival (95% confidence interval) while remaining on hemodialysis

	Number	5 years	10 years	15 years
All patients (primary outcome)	1780	91.6 (89.8–93.4)	81.4 (77.9–85.0)	70.9 (65.1–77.2)
by spKt/V				
≤ 1.4	489	85.0 (79.6–90.7)	69.3 (59.4–80.9)	56.1 (39.2–30.3)
> 1.4–1.6	531	94.1 (91.5–96.9)	83.0 (76.8–89.8)	76.3 (67.9–85.8)
> 1.6	759	92.2 (89.9–94.7)	84.0 (79.6–88.5)	72.7 (65.3–81.0)
Complete–cases^1^	1493	91.3 (89.4–93.2)	80.6 (76.9–84.4)	71.4 (65.7–77.6)
Weibull null-model estimates		91.4	81.0	70.7

^1^Used for estimation of adjusted hazard ratios and secondary outcome analysis in the Weibull regression model

**Fig. 2:**
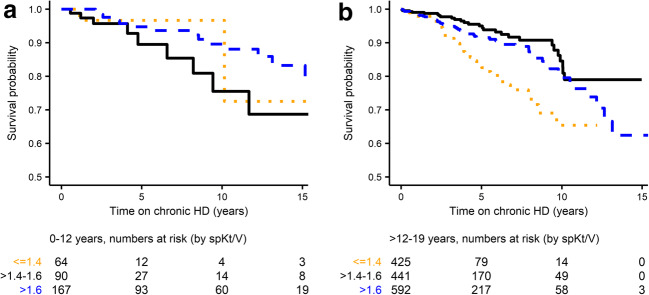
Survival while remaining on chronic hemodialysis stratified by spKt/V within age groups (Kaplan–Meier curve). **a** 0–12 years at the start of dialysis; log-rank test target (solid) *versus* high (dashed) spKtV (> 1.4–1.6 *versus* > 1.6): *P* = 0.2 (ns). **b** > 12–19 years at the start of dialysis; log-rank test low (dotted) *versus* target (solid) spKtV (≤ 1.4 *versus* > 1.4–1.6): *P* < 0.001; and target (solid) *versus* high (dashed) spKtV (> 1.4–1.6 *versus* > 1.6): *P* = 0.09 (ns)

Within the group of patients starting HD at 0–12 years of age (Fig. 2A), lower survival was suggested with target spKt/V > 1.4–1.6 compared to high spKt/V > 1.6, but this was not statistically significant (*P* = 0.2). No further comparisons were made with low spKt/V < 1.4 due to small patient numbers. For the group of patients starting HD > 12–19 years (Fig. 2B), low spKt/V < 1.4 was associated with lower survival compared to target spKt/V > 1.4–1.6 (*P* < 0.001) and high spKtV > 1.6 (*P* = 0.004). There was no significant difference between high spKtV > 1.6 and target spKt/V > 1.4–1.6 (*P* = 0.09). The subgroup analysis performed within age group ≤ 12 years did not show statistical significance of mean spKt/V within the subgroups < 6 years and 6–12 years at the start of HD (Online Resource Fig. S1).

A Weibull model captured the form of the baseline hazard well, with 5-, 10- and 15-year predictions within the 95% confidence interval of the Kaplan–Meier curve (red line, Online Resource Fig. S2A). The corresponding estimated scale and shape parameters of the baseline hazard function were σ = 35.4 years (time when predicted survival = 40%) and α = 1.23 (indicating with α > 1 increasing hazard of death over time). The estimated hazard ratio (HR) from the unadjusted Weibull proportional hazards model indicated an increased risk for low versus target spKt/V (HR = 2.3, 95%CI: [1.4–3.6], *P* < 0.001), but there was no discernable difference between target and high spKt/V (HR = 1.05, [0.69–1.61], *P* = 0.804). These estimates did not alter significantly (HR = 2.3 for low versus target spKt/V, *P* < 0.001; HR = 1.2 for high versus target spKt/V, *P* = 0.44) following adjustment for age (HR = 0.66 for < 12 years compared to 12–19 years, *P* = 0.09), etiology (HR = 2.5 for secondary glomerulonephritis, *P* < 0.001; other etiologies did not show significant associations) and ethnicity (HR = 1.5 for African origin, *P* = 0.025). An interaction of spKt/V with age was observed that was significant for high spKt/V > 1.6 (HR = 0.32 for age < 12 years, *P* = 0.03), but not in the reference group treated with target spKt/V (*P* = 0.35) or in the low dose group ≤ 1.4 (*P* = 0.19) (Online Resource Fig. S3).

The association shown in Fig. 1 did not change when including patients only up to 19 years of age in the analysis (Online Resource Fig. S4A). Investigation of Kaplan–Meier curves on log(time) versus log(-log(S(t))) scale (Online Resource Fig. S2B) and visual inspection of the Schoenfeld residuals did not indicate any clear deviation from proportional hazard assumption. Nevertheless, the post hoc sensitivity analysis considering piece-wise survival showed that spKt/V was not significantly associated with survival within the first 2 years (adjusted HR = 1.9 for low versus target spKt/V, *P* = 0.18). The estimated adjusted HR for the subgroup of patients with follow-up of at least 2 years (*n* = 975) was 2.4 (*P* = 0.002) for low versus target spKt/V.

### Secondary outcome

**Fig. 3:**
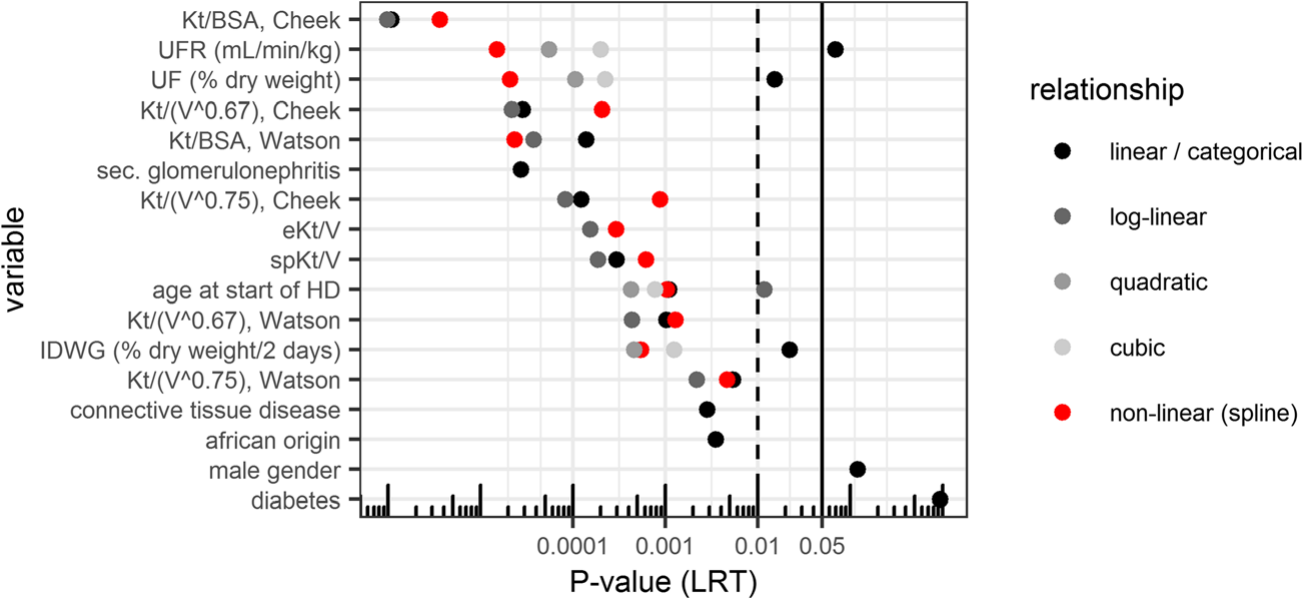
Summary of monovariate relationships tested, ordered by relative importance of each variable (lowest *P* value according to likelihood ratio test, LRT) for the best fitting relationship. *Black solid line*: *P* = 0.05, *black dashed line*: *P* = 0.01

**Fig. 4:**
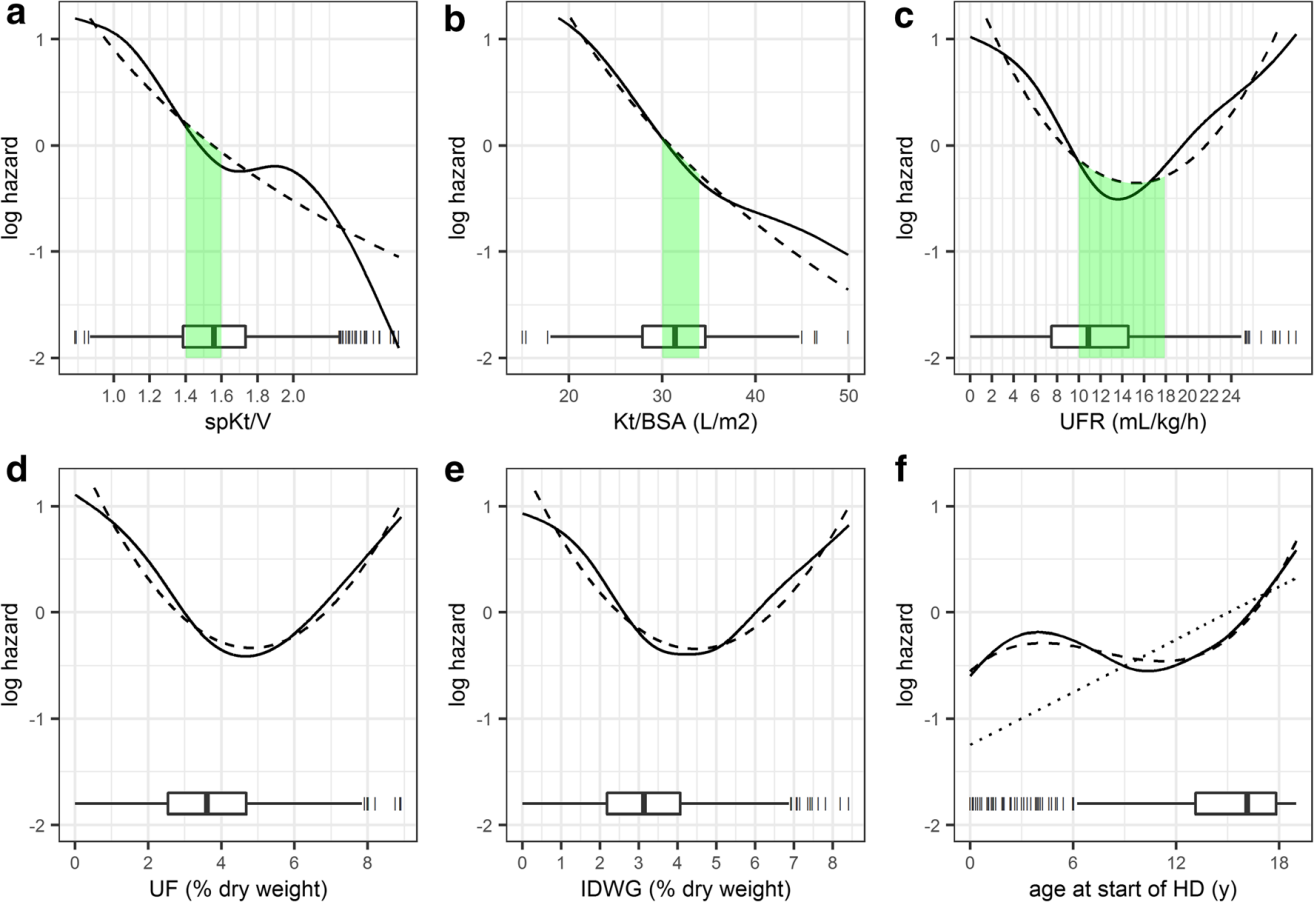
Illustration of predicted log-hazard of monovariate models (centered to mean value of each presented covariate). *Solid line*: Prediction from flexible nonlinear spline model. *Dashed line*: Prediction from more simple parametrizations (Kt/BSA and spKt/V: log-linear relationship; ultrafilration and interdialytic weight gain (IDWG): quadratic relationship; age: linear/cubic relationship (*dashed line*: cubic, *dotted line*: linear)). The variable distribution is illustrated in the bottom of each panel by standard boxplots, 3 values of spKt/V and Kt/BSA beyond limits of the panel are not illustrated for better visualization. *Green shaded areas*: spKt/V reference target of 1.4–1.6, approximately corresponding Kt/BSA of 30–34 L/m^2^ (see Fig. 5). UFR of 10–18 mL/kg/h associated with low harzard in both spline and simpler parametric model.

The association of investigated covariates with absolute survival from the Weibull accelerated failure time (AFT) regression model is shown in Fig. 3, ordered by covariate importance (*P* value of LRT). Kt/BSA showed a stronger association with survival (*P* < 0.001, linear and log-linear relationships, using V_TBW_ according to Cheek et al. [23, 24]) compared to other metrics of dialysis adequacy, and in particular, compared with spKt/V and eKt/V (*P* < 0.001, log- and linear relationship). Indices of fluid balance were nonlinearly related with survival; the strongest association was found for UFR (*P* < 0.001, quadratic relationship). Age at the start of dialysis was also associated with survival (*P* < 0.001, cubic relationship; *P* = 0.001, linear relationship). The relationship between these variables and model-predicted log-hazard is illustrated in Fig. 4. No interaction with age was apparent for Kt/BSA or UFR. Among further baseline demographic factors, etiology (secondary glomerulonephritis) was most strongly associated with lower survival (*P* < 0.001), followed by age at the start of dialysis, connective tissue disease (*P* = 0.003), and African origin (*P* = 0.004). No association could be found for gender (*P* = 0.12) and diabetes as comorbidity (*P* = 0.94). In the sensitivity analysis (censoring patients at 19 years of age) similar associations were observed, while suggesting that the use of the Cheek equation (and not Watson) is particularly relevant < 19 years of age for the strong log-linear relationship with Kt/BSA observed (Online Resource Fig. S4B).

**Fig. 5:**
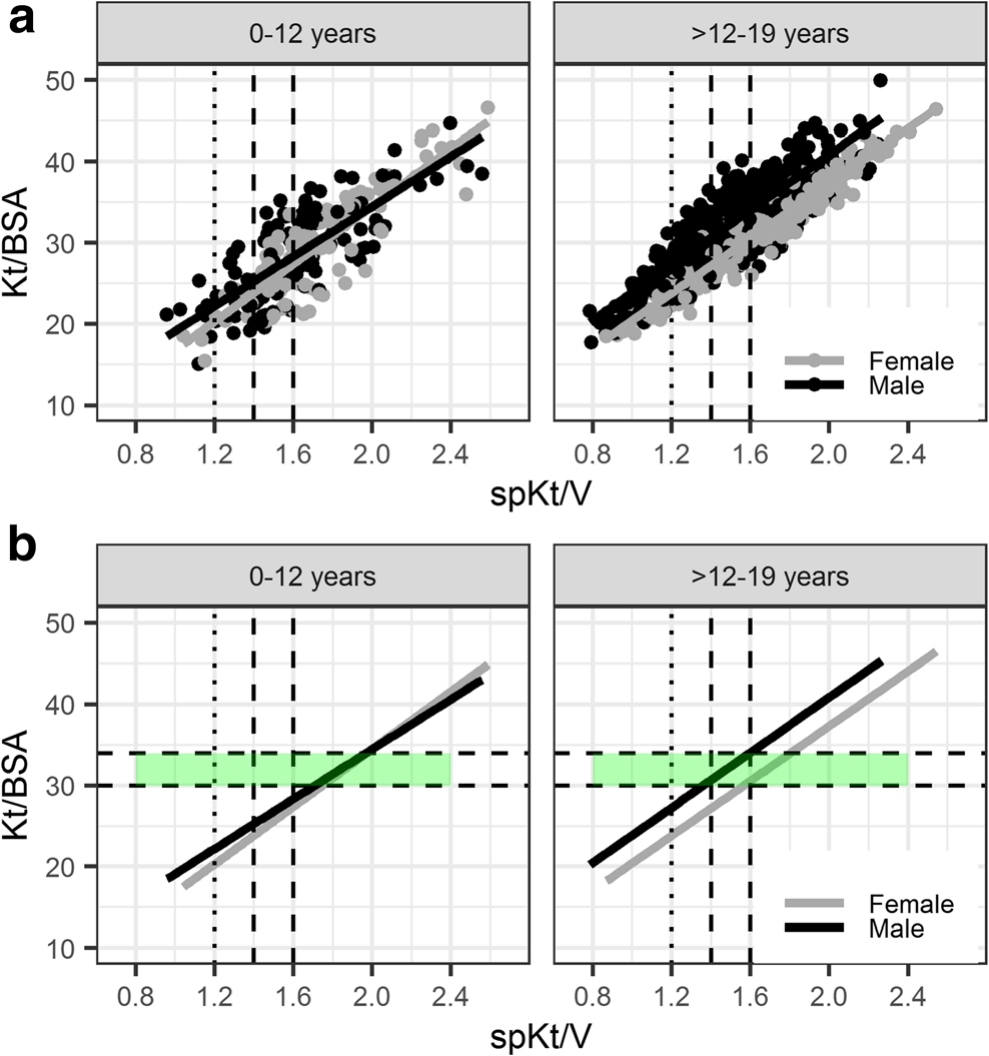
Correlation between spKt/V and Kt/BSA stratified by age at the start of HD and gender (**a**: data with linear regression line illustrated, **b**: only linear regression line illustrated): Kt/BSA translates to higher spKt/V in younger (0–12 years) and female patients (> 12–19 years), thus there is a risk of underdosing HD in these patients if same spKt/V is targeted for all. *Dotted vertical line*: minimum spKt/V of 1.2 targeted in adults. *Dashed vertical lines:* target spkt/V of > 1.4–1.6. *Horizontal dashed lines with green shaded area*: Kt/BSA of 30–34 L/m^2^, corresponding approximately to spKtV of 1.4–1.6 in adolescent boys, 1.6–1.8 in adolescent girls, and > 1.6 in children 0–12 years (≥ 1.8 in the subgroup of children < 6 years, data not shown separately).

**Fig. 6:**
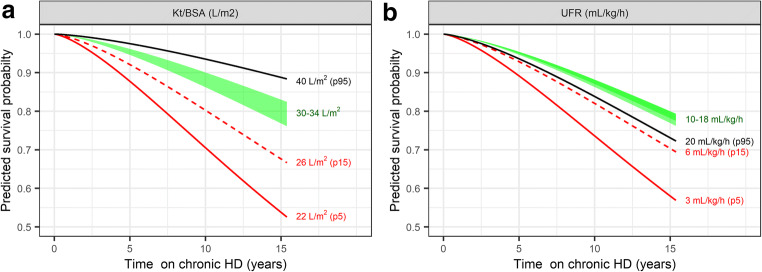
Predicted survival from the fitted multivariate adjusted Weibull AFT model for different Kt/BSA and UFR for a mean reference patient of the population (15 years at the start of HD, etiology not secondary glomerulonephritis/vasculitis and ethnic origin not African American). *Green shaded area*: Kt/BSA of 30–34 L/m^2^ (percentiles p38 to p71 in the population, see Fig. 4), corresponding approximately to spKtV of 1.4–1.6 in adolescent boys, 1.6–1.8 in adolescent girls and > 1.6 in children < 12 years (see Fig. 5). UFR of 10–18 mL/kg/h (percentiles p43 to p92 in the population, see Fig. 4) is associated with lower risk in parametric models.

In the multivariate analysis, Kt/BSA (log-linear), UFR (quadratic), age (linear), etiology, and ethnic origin were statistically significant following forward and backward selection (Estimates: Online Resource Table S1A). Parameter estimates for a cubic age relationship could not be estimated with good precision (Online Resource Table S1B) but provided better model predictions as evaluated by comparison with Kaplan–Meier plots. No interaction with age at the start of HD could be found. Correlation between spKt/V and Kt/BSA stratified by age and gender is shown in Fig. 5A, illustrating that Kt/BSA of 30–34 L/m^2^ would correspond approximately to spKtV of 1.4–1.6 in adolescent boys, 1.6–1.8 in adolescent girls, and > 1.6 in children < 12 years (≥ 1.8 in the subgroup of children < 6 years) (Fig. 5B). Predicted survival curves for the final multivariate model are illustrated for different Kt/BSA and UFR values for a mean patient (15 years at the start of HD, etiology not secondary glomerulonephritis/vasculitis, ethnic origin not African American) in Fig. 6.

Correlations with previously identified disease-related mortality risk factors are depicted in Online Resource Fig. S5, showing no major correlation with main risk factors (maximal correlation of ρ = 0.12 for albumin and ρ = –0.28 for lactate dehydrogenase with Kt/BSA, ρ = 0.09 for red blood cell distribution width with UFR).

## Discussion

This retrospective observational study is the first to demonstrate an association between low small solute clearance (spKt/V < 1.4) and a higher risk of mortality on long-term HD in patients having started HD in childhood (0–19 years, median: 16 years). To our knowledge, this patient cohort is uniquely the largest pediatric cohort studied receiving standardized HD from the same provider, allowing detailed analysis of all HD treatment and laboratory data over time. There is limited data on survival for pediatric patients remaining on HD for more than 10 years with which to compare our results. Our 5-year survival rate of 91.6% may however be considered consistent with a previously reported 4-year survival rate of 92.3% in pediatric patients starting HD between 2007–2011 [28]. Compared to patients treated with target spKt/V > 1.4–1.6, survival on chronic HD was 9–20% lower after 5–15 years, respectively. Additional benefit of high spKt/V > 1.6 was suggested in patients starting HD < 12 years of age, but not in those starting HD as adolescents. Interestingly, the apparent age-dependent dose–response relationship disappeared when considering Kt/BSA (using pediatric V_TBW_ calculated according to Cheek et al. [23, 24] for calculation) as the dialysis adequacy metric. Similar to prior reports in adults, Kt/BSA further showed a stronger association with survival than spKt/V [29]. Previously not reported, fluid balance indices in our study showed a strong association with survival, with both low and very high UFR associated with an increased risk of mortality in this young population. Specifically, no clearly increased mortality risk was observed in the range of 10–18 mL/kg/h (lowest risk in the range of 13–15 approximately), in contrast to observational findings in adults [6].

In adults, the increased mortality risk with UFR > 10–13 mL/kg/h is possibly explained by higher incidence of intradialytic hypotension and associated risk of organ ischemia [28]. Increased UFR was not associated with reduced intradialytic blood pressure in our population (lowest intradialytic systolic blood pressure values rather tended to be higher in patients treated with high UFR; data not shown) suggesting that most patients hemodynamically tolerated high UFR. The frequent use of high UFR in our population [14] may hence reflect that children and young adults having started HD during childhood require higher fluid intake to meet nutritional needs and promote growth, and/or that they have better tolerance of high UFR. Increasing UFR for dry weight optimization can improve anemic and inflammatory control [29], with the added benefit of increased convective clearance of larger uremic solutes associated with cardiovascular disease and mortality [30]. The benefit of actively limiting UFR, especially without increasing treatment duration, has been questioned in adults: epidemiologically a 15% decrease in mean UFR during the recent years has not resulted in reduced cardiovascular hospitalization rates [31]. Potentially worrisome, we observed a 40% decrease in UFR during our study period (from mean 12.2 to 7.3 mL/kg/h between 2004 and 2015, data not shown) in our population, while spKt/V prescriptions remained stable over time.

The association of low small solute clearance with a higher risk of mortality persisted after adjustment for baseline characteristics, including etiology of kidney disease (secondary glomerulonephritis), ethnicity (African origin), and older age at the start of HD. Connective tissue disease as a comorbidity was also associated with mortality in univariate analyses but not in multivariate analysis, probably due to its correlation with secondary glomerulonephritis/vasculitis. In contrast to previous reports [20], we could not demonstrate a significant association of mortality with female gender (though a trend toward better outcomes for male patients was observed in univariate analysis) or diabetes as comorbidity.

The relationship between age and survival on a continuous scale showed a relatively high mortality risk in those starting HD at 2–5 years of age, compared to those starting around 12 years of age, in line with the literature [20]. Within the adolescent age-group, we observed a risk increase with age, which is also consistent with USRDS data reporting higher mortality in patients initiating dialysis as young adults compared to children 0–19 years [21].

Evaluating spKt/V–age interactions (Fig. 2 and Fig. S3) indicated that decreasing survival in adolescents may be confounded by a larger proportion of adolescents being treated with below-target doses (spKt/V ≤ 1.4) as compared to patients starting HD <12 years of age, while not benefitting from high spKt/V > 1.6 as opposed to younger patients. We have previously reported age-related differences in dialysis prescription in this population [19], in line with others [12], where we further found adolescents ≥ 100 kg of particular risk to receive low spKt/V [19]. The lack of benefit of higher spKt/V > 1.6, particularly in adolescents, may be related to the different weight–BSA relationship (and hence spKt/V–Kt/BSA relationship). This has been pointed out by others [12, 13], and may be compensated by benefits of longer treatment duration in obese adolescents (potentially associated with improved treatment of overhydration and clearance of time-dependent solutes) [30]. Interestingly, our spKt/V–Kt/BSA relationship observed in adolescents is consistent with the relationship for adults [13], while Kt/BSA is, as expected, lower in younger patients for a given spKt/V. We found that Kt/BSA of > 30 L/m^2^ corresponded approximately to spKtV of > 1.4 in adolescent boys (as reported for male adults [13]), > 1.6 in adolescent girls (in line with female adults [13]) and children < 12 years (≥ 1.8 in children < 6 years, data not shown separately). This observation may also support the finding that adult women benefit from higher spKt/V compared to men [5].

Results are consistent with a previous exploratory machine-learning based analysis on a subgroup of patients [27], suggesting optimal spKt/V > 1.5 and UFR > 10 mL/kg/h for an average adolescent patient. In the present analysis, we did not investigate relationships of survival with other disease-related risk factors of mortality, such as albumin, lactate dehydrogenase, and red blood cell distribution width. In our supplementary analysis, we could not identify strong correlations with those variables, suggesting little risk of confounding by those variables. It also suggests that those disease-related variables are only marginally affected by HD treatment, stressing the importance of other intervention strategies to improve patient survival and well-being, e.g., related to treatment of nutrition and anemia. Some stronger correlation with z-score weight for age and normalized protein catabolic rate with investigated treatment-related variables could be observed. This deserves further investigation, as we have observed suboptimal growth particularly in patients < 12 years of age [19], and as others have reported improved growth in children treated with more intense (longer or more frequent) dialysis [31, 32].

As we report on observational data, shown associations must be interpreted with caution regarding causality. It may be emphasized in this context that presented survival probabilities refer to “survival on chronic HD”, and not to “overall survival”, as no information on survival after kidney transplantation or facility discharge was available. As a cross check, we also performed a post hoc supplementary analysis taking into account transplantation as a competing risk (instead of random censoring). The results were consistent with the primary analysis, albeit the long-term survival probability was higher than in the presented analysis considering transplantation as a censoring event (Online Resource Fig. S6). We did not further investigate the influence of intraindividual variation in UFR or spKt/V prescription, which was higher for UFR (average intraindividual variation: ±48% or ±5 mL/kg/h) than for spKt/V (±15% or ±0.24). Proposed Kt/BSA reference ranges may not apply to children and infants < 2 years, in whom BSA-normalized healthy glomerular filtration rate is lower than in older patients [33]. Our ability to investigate age-dependent associations was partly limited by small patient numbers < 12 years (and especially < 6 years) of age. The trend toward lower association with spKt/V during the first two years of HD could be related to residual kidney function (RKF). RKF was not evaluated systematically in all patients but was reported only in a small percentage of patients (4%) [19]. It is unlikely to have a significant effect on our long-term results as RKF tends to be lost in the majority of patients within the first year of starting HD on a thrice-weekly regimen [34]. In such cases, we would however expect better survival than observed in those patients. Although relatively high UFR in children of 10–18 ml/kg/h appears to be risk-free regarding their survival, that may only be true if clinically well tolerated (as suggested from its frequent use in the presented population in the outpatient setting [14]), if associated with improved nutrition (as suggested from positive correlation of ρ = 0.41 between UFR and normalized protein catabolic rate in Fig. S4), and if necessary, to achieve or optimize dry weight. Potentially negative (i.e., myocardial stunning) or positive (i.e., decreased chronic fluid retention) effects of high UFR on the cardiovascular system could not be assessed in our study. Previously recommended and historically well-tolerated fluid removal rate in children is reported to be up to 8% of body weight [35]. If 18 mL/kg/h UFR is used, in a 25 kg child, 1350 mL of fluid is removed in a 3 h HD session, corresponding to ~5.4% of body weight. This is well under 8%, supporting our finding that UFR of 10–18 ml/kg/h appears risk free. As patients were treated in > 1000 different clinical centers, we cannot exclude influence of regional differences in patient care [36], potentially limiting generalizability of results to regions other than the US. However, all patients uniformly received treatment by the same dialysis provider, which resulted in standardized delivery, evaluation, and recording of HD treatment. This allowed us to perform an analysis of a uniquely large paediatric cohort over an extended period. As our analysis shows that mortality increases over time on dialysis, consistent with previous reports [37], the ultimate goal of early kidney transplantation remains undisputed.

In conclusion, observational findings from this first study in patients starting maintenance HD in childhood demonstrate the benefit of achieving spKt/V > 1.4. Kt/BSA appears to be a clinically more meaningful marker of HD adequacy compared to spKt/V, supporting targeting higher spKt/V in younger children. In children and adolescents requiring long-term hemodialysis, higher UFR of 10–18 mL/kg/h was associated with best survival, in contrast to data reported in adults. With the goal to improve long-term outcomes in pediatric HD, we suggest that original findings reported here serve as guidance in HD dose and UFR prescription.

## Supplementary information

ESM 1 (PDF 558 kb).

## Data Availability

The deidentified data used is owned by DaVita and cannot be shared by the authors.

## Code availability

Regression models are shared in detail in the Supplementary data with an example R-code that can be used for the Weibull model simulations.
