# Supplementary material for: Hemodialysis (HD) dose and ultrafiltration rate are associated with survival in pediatric and adolescent patients on chronic HD—a large observational study with follow-up to young adult age
Source: Pediatr Nephrol. 2021 Mar 2;36(8):2421–32. doi: 10.1007/s00467-021-04972-6 (PMC8260402; doi:10.1007/s00467-021-04972-6)
Supplement: Supplementary file 1 — (PDF 558 kb). [file 467_2021_4972_MOESM1_ESM.pdf]

Online Resource (Supplemental Material)

Hemodialysis (HD) dose and ultrafiltration-rate are associated with survival in pediatric and adolescent patients on chronic HD

V Gotta (1), O Marsenic (2), A Atkinson (1), M Pfister (1,3)

(1) Pediatric Pharmacology and Pharmacometrics, University of Basel Children's Hospital, Basel Switzerland
(2) Pediatric Nephrology, Stanford University School of Medicine, Lucile Packard Children's Hospital, Stanford, CA
(3) Certara, Princeton, NJ, USA

Corresponding author: Verena Gotta, verena.gotta@ukbb.ch

Table of Contents

Figure S1: Subgroup analysis of patients having started HD <= 12 years of age. **A**: patients having started <6 years, **B**: patients having started 6-12 years. *Page 2*

Figure S2: **A**: Unadjusted Weibull model survival prediction (red dashed line) versus non-parametric survival curve (black line, 95% confidence interval depicted by grey shaded area). **B**: Investigation of Kaplan-Meier curves on log(time) vs log(-log(survival(t))) scale of the primary outcome. *Page 3*

Figure S3: Estimated hazard ratios (HR) from the adjusted Weibull proportional hazards regression model for the primary outcome. *Page 4*

Figure S4: Sensitivity analysis of primary outcome (A) and secondary outcome (B): censoring all patients at 19 years of age. *Page 5*

Figure S5: Correlation of main treatment-related variables with laboratory ("disease-related") variables found to be related with mortality in previous machine learning analysis. *Page 6*

Figure S6: **A**: Comparison of Kaplan-Meier plot of probability of survival on chronic HD versus its corresponding cumulative incidence function estimated treating transplantation as competing risk. **B**: Primary outcome estimates (survival on chronic HD stratified by spKt/V) treating transplantation as competing risk. *Page 7*

Table S1: Parameter estimates of the fitted multivariate accelerated failure time (AFT) Weibull model. **A**: using a linear relationship with age. **B**: using a cubic relationship with age *Page 8*

Supplementary Data: Example R-code for Weibull model simulations. *Page 9*

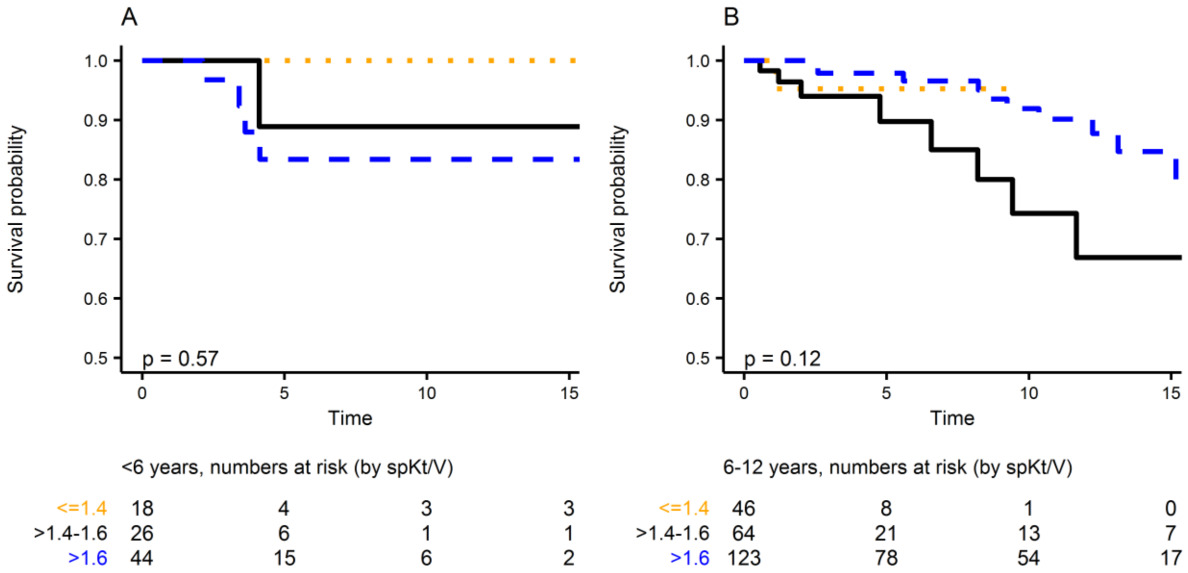

Figure S1: Subgroup analysis of patients having started HD <= 12 years of age. **A**: patients having started <6 years, **B**: patients having started 6-12 years.

A

B

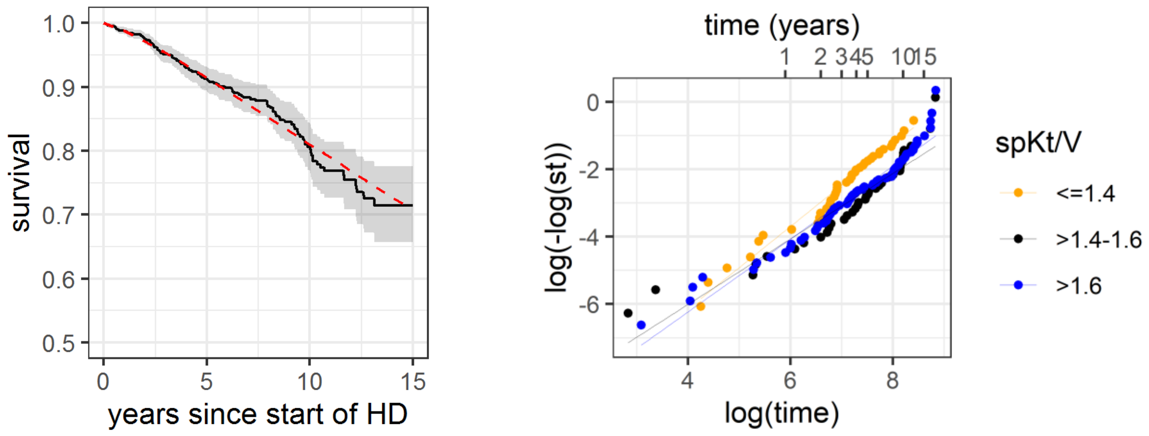

Figure S2: **A**: Unadjusted Weibull model survival prediction (red dashed line) versus non-parametric survival curve (black line, 95% confidence interval depicted by grey shaded area). **B**: Investigation of Kaplan-Meier curves on log(time) vs log(-log(survival(t))) scale of the primary outcome.

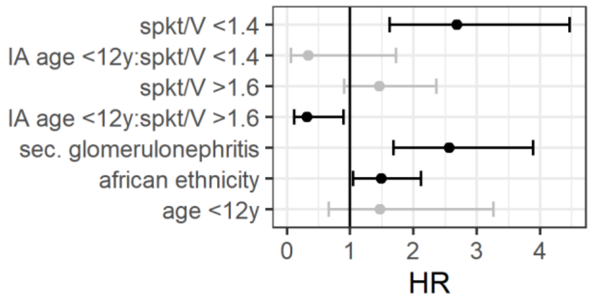

Figure S3: Estimated hazard ratios (HR) from the adjusted Weibull proportional hazardsregression model for the primary outcome (survival by spKtV: target>1.4-1.6 = reference with HR=1 (vertical line) *versus* spKtV≤1.4 and spKtV>1.6, HR >1 indicating increased risk of mortality) including an interaction (IA) term between spKt/V and age (HD start >12-19 years = reference with HR=1). *Horizontal lines*: 95% confidence intervals. *Sec. GN*=secondary glomerulonephritis/vasculitis as aetiology of renal disease.

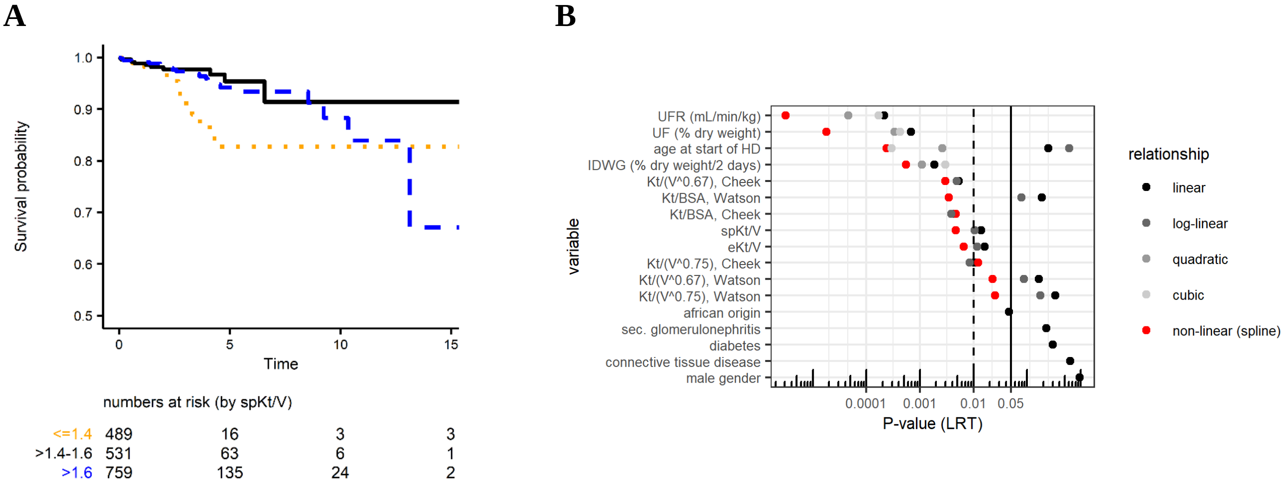

Figure S4: Sensitivity analysis of primary outcome (A) and secondary outcome (B): censoring all patients at 19 years of age. **A**: Survival probability *up to 19 years of age* while remaining on chronic hemodialysis (Kaplan-Meier-curve) by mean spKt/V delivered; log-rank test: spKtV ≤1.4 (dotted) *versus* >1.4-1.6 (solid): P=0.02. spKtV <1.4 versus >1.6 (dahsed): P=0.02. **B:** Summary of monovariate relationships tested, ordered by relative importance of each variable (lowest P-value according to likelihood ratio test, LRT) for the best fitting relationship. *Black solid line*: P=0.05, *black dashed line*: P=0.01.

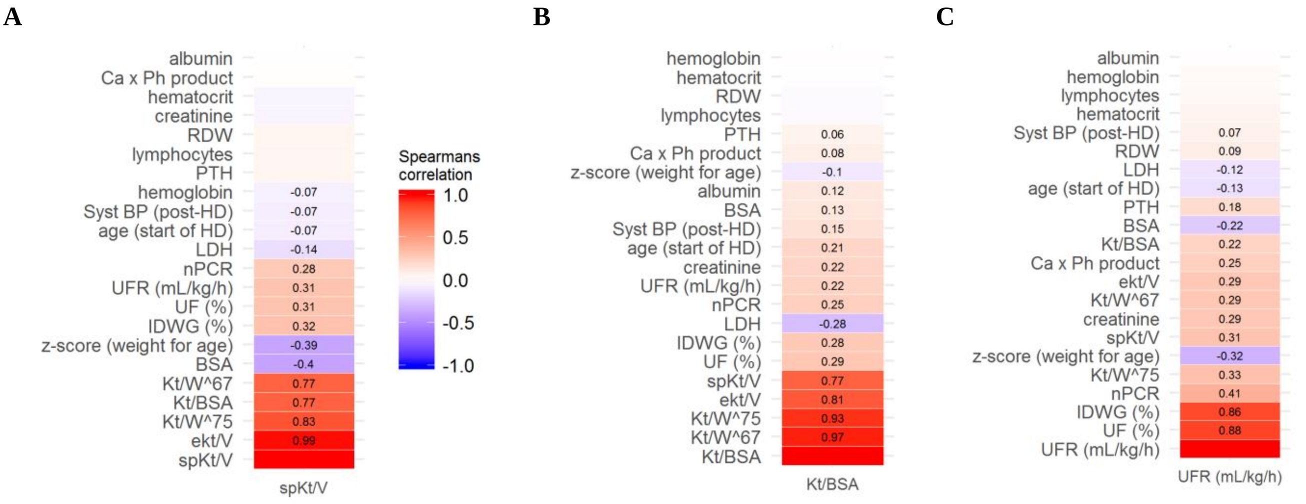

Figure S5: Correlation of main treatment-related variables with laboratory ("disease-related") variables found to be related with mortality in previous machine learning analysis (Gotta V & Tancev G et al. NDT 2020). **A**: correlation with spKt/V. **B**: correlation with Kt/BSA. **C**: correlation with ultrafiltration rate (UFR). Red colour indicates positive correlation, blue colour negative correlation. Every panel is ordered by magnitude of correlation with variables on x-axis. Only correlations with p-value <0.05 are numerically depicted. Abbrevations: Ca x Ph product: calcium-phosphate product (albumin corrected). RDW: red blood cell distribution width. PTH: parathomone. Syst BP: systolic blood pressure. LDH: lacate dehydrogenase. nPCR: normalized protein catabolic rate. UFR: ultrafiltration rate. UF (%): total ultrafiltration in % of target dry weight. IDWG: inter-dialytic weight gain.

A **B**

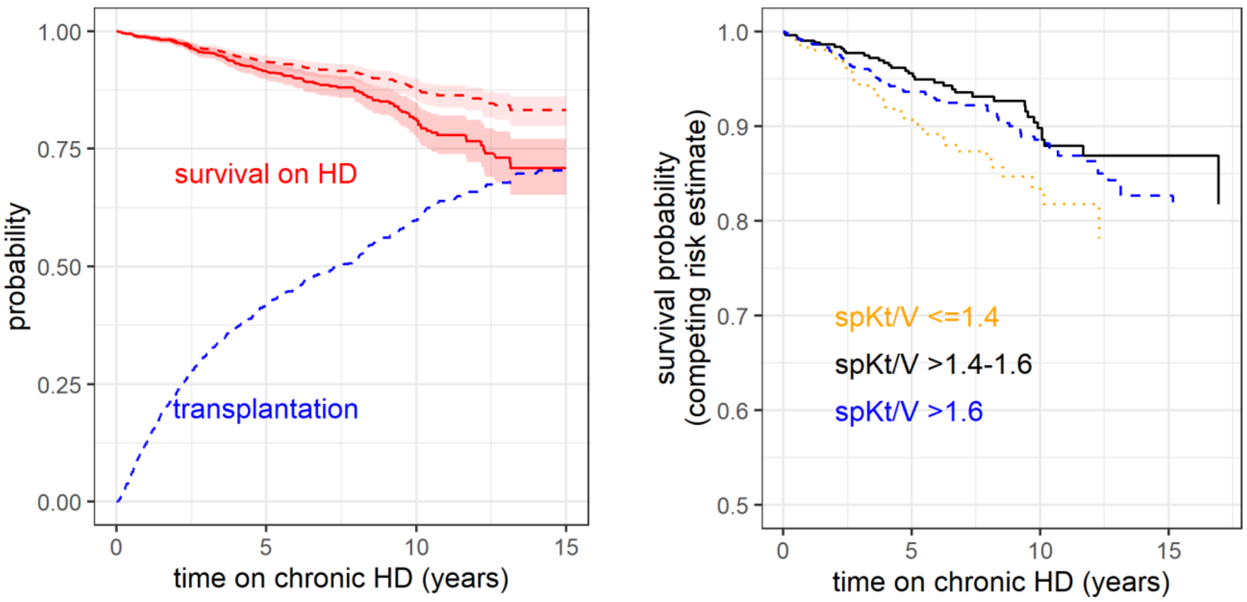

Figure S6: **A**: Comparison of Kaplan-Meier plot of probability of survival on chronic HD (*red solid line*) versus its corresponding cumulative incidence function (*red dashed line*) estimated treating transplantation (*blue dashed line*) as competing risk. Shaded areas: 95% confidence intervals **B**: Primary outcome estimates (survival on chronic HD stratified by spKt/V) treating transplantation as competing risk. Test for equality across groups: P=0.04. Pairwise comparisons: low (≤1.4) versus target (>1.4-1.6) spkt/V: P=0.01, low (≤1.4) versus high (>1.6): P=0.12, target (>1.4-1.6) versus high (>1.6): P=0.19.

Table S1: Parameter estimates of the fitted multivariate accelerated failure time (AFT) Weibull model

A: using a linear relationship with age

**Parameter** (relationship)	**AFT Estimate**	**Standard error**	**P-value**	univariate AFT estimate
Kt/BSA (log-linear)				
$\gamma(_{Log(kt/BSA\ /\ mean)})$	1.95	0.43	<0.001	2.16
UFR (quadratic)				
$\gamma(_{UFR-mean})$	0.15	0.04	<0.001	0.19
$\gamma(_{UFR^2-mean^2})$	-0.0057	0.0013	<0.001	-0.0063
Age (linear)				
$\gamma_{(age-mean)}$	-0.063	0.019	<0.001	-0.064
Categorical variables				
$\gamma_{sec.glomerulonephritis}$	-0.62[a]	0.15	<0.001	-0.76
$\gamma_{african}$	-0.32[b]	0.13	0.015	-0.42
Baseline hazard*				
Log(scale)<br>Weibull α [-]	-0.35<br>1.42	0.07	NA	-0.21 (null model)<br>1.23
Intercept<br>Weibull σ=1/λ [years]	9.7<br>40.7	0.16	NA	9.5 (null model)<br>35.4

[a]corresponding to a hazard ratio (HR) of 2.4 in a Weibull proportional hazard model; [b]corresponding to a HR of 1.6 in a Weibull proportional hazard model.

B: using a cubic relationship with age.

**Parameter** (relationship)	**AFT Estimate**	**Standard error**	**P-value**	univariate AFT estimate
Kt/BSA (log-linear)				
$\gamma(_{log(kt/BSA)-log(\ mean)})$	1.93	0.43	<0.001	2.16
UFR (quadratic)				
$\gamma(_{UFR-mean})$	0.16	0.04	<0.001	0.19
$\gamma(_{UFR^2-mean^2})$	-0.0058	0.0013	<0.001	-0.0063
Age (cubic)				
$\gamma_{(age-mean)}$	-0.25	0.21	0.234	-0.11
$\gamma_{(age^2-mean^2)}$	0.028	0.021	0.197	0.018
$\gamma_{(age^3-mean^3)}$	-0.001	0.0007	0.13	-0.0008
Categorical variables				
$\gamma_{sec.glomerulonephritis}$	-0.59	0.15	<0.001	-0.76
$\gamma_{african}$	-0.32	0.13	0.012	-0.42
Baseline hazard*				
Log(scale)<br>Weibull α [-]	-0.35<br>1.42	0.07	NA	-0.21 (null model)<br>1.23
Intercept<br>Weibull σ=1/λ [years]	9.7<br>40.7	0.16	NA	9.5 (null model)<br>35.4

* reference values: mean Kt/BSA of 31.3 L/m2, mean UFR of 11.08 L/kg/h, mean age of 15.08 years, aetiology not secondary glomerulonephritis/vasculitis, ethnicity not African origin. Calculation of baseline Weibull distribution parameters: α = 1/scale, σ=1/λ (with λ = exp(-intercept).

Supplementary Data: Example R-code for Weibull model simulations

```
mktBSA <- 31.3      # mean Kt/BSA
mUFR <- 11.1        # mean UFR

mu0.hat <-   9.7    # intercept
lambda0.hat <- exp(-mu0.hat)

sigma.hat <- exp(-0.35) # scale
alpha.hat <- 1/sigma.hat

# time vector
tt.vec <- 0:(15*365)

# reference prediction
surv0.vec <- 1-pweibull(tt.vec, shape=alpha.hat, scale=1/lambda0.hat)


# coefficient relating to log(Kt/BSA)
coef_logBSA <- 1.93

# prediction for Kt/BSA=22 L/m2
gamma.hat.ktBSA_22 <- logBSA*log(22/mktBSA)
surv.vec.ktBSA_22 <- surv0.vec^(exp(-gamma.hat.ktBSA_22/sigma.hat))

pred.tab.ktBSA <- data.frame(time_days = tt.vec,
                          time_years = tt.vec/356,
                          surv_ref = surv0.vec,
                          surv_ktBSA_22 = surv.vec.ktBSA_22)



# coefficients relating to UFR
coef_ufr_lin <- 0.15
coef_ufr_sq <- -0.0057

# prediction for UFR=10 ml/kg/h
gamma.hat.ufr_10_lin <- coef_ufr_lin *(10- mUFR)
gamma.hat.ufr_10_sq <- coef_ufr_sq *(10**2-mUFR**2)

surv.vec.ufr_10 <- surv0.vec^(exp(-(gamma.hat.ufr_10_lin+gamma.hat.ufr_10_sq)/sigma.hat))


pred.tab.ufr <- data.frame(time_days = tt.vec,
                             time_years = tt.vec/356,
                             surv_ref = surv0.vec,
                             surv_ufr_10 = surv.vec.ufr_10)

# plot predictions
library(ggplot2)

ggplot(pred.tab.ufr, aes(time_years, surv_ref)) +
  geom_line(col="black", size=0.1)+
  geom_line(aes(time_years, surv_ufr_10), col="red")+
  scale_y_continuous("predicted survival", limits=c(0.5, 1)) +
  scale_x_continuous("time (years)")

ggplot(pred.tab.ktBSA, aes(time_years, surv_ref)) +
  geom_line(col="black", size=0.1)+
  geom_line(aes(time_years, surv_ktBSA_22), col="red")+
  scale_y_continuous("predicted survival", limits=c(0.5, 1)) +
  scale_x_continuous("time (years)")
```
